# Wildfire-related PM_2.5_ and respiratory transmitted disease among Chinese children and adolescents from 2008 to 2019: A retrospective study

**DOI:** 10.1371/journal.pmed.1004613

**Published:** 2025-12-05

**Authors:** Li Chen, Rongbin Xu, Junqing Xie, Yi Xing, Bo Wen, Yao Wu, Binbin Su, Mengjie Geng, Xiang Ren, Yi Zhang, Jieyu Liu, Xinli Song, Yang Qin, RuoLin Wang, Jianuo Jiang, Tongjun Guo, Wen Yuan, Yinghua Ma, Yanhui Dong, Yi Song, Jun Ma, Shanshan Li, Yuming Guo

**Affiliations:** 1 Institute of Child and Adolescent Health, School of Public Health, Peking University; National Health Commission Key Laboratory of Reproductive Health, Beijing, China; 2 UNESCO Chair on Global Health and Education of Peking University, Beijing, China; 3 Chongqing Emergency Medical Center, Chongqing University Central Hospital, School of Medicine, Chongqing University, Chongqing, China; 4 Climate, Air Quality Research Unit, School of Public Health and Preventive Medicine, Monash University, Melbourne, Australia; 5 Centre for Statistics in Medicine, NDORMS, University of Oxford, Oxford, United Kingdom; 6 School of Population Medicine and Public Health, Chinese Academy of Medical Sciences/Peking Union Medical College, Beijing, China,; 7 Division of Infectious Disease Control and Prevention, Key Laboratory of Surveillance and Early Warning on Infectious Disease, Chinese Center for Disease Control and Prevention, Beijing , China; The Hospital for Sick Children, CANADA

## Abstract

**Background:**

Exposure to fine particles (PM_2.5_) from wildfires is known to cause deaths and chronic diseases, but its effect on respiratory infections, especially in children and adolescents, is not well characterized. We aimed to comprehensively assess the association between short-term exposure to wildfire-related PM_2.5_ and the incidence and mortality of respiratory transmitted diseases in children and adolescents.

**Methods and findings:**

Data on daily counts of incident and mortality cases of respiratory transmitted diseases in persons aged 4–24 years old were collected from China Information System for Disease Control and Prevention, covering 501 cities from 2008 to 2019. Daily concentrations of wildfire-related PM_2.5_ were estimated using machine learning and chemical transport models at a 0.25°×0.25° spatial resolution. We used time-stratified case-crossover design with conditional logistic regression to estimate the association between short-term exposures to wildfire-related PM_2.5_ and incidence and mortality of respiratory transmitted diseases, adjusting for temperature, relative humidity, precipitation, and total PM_2.5_. There were 6,089,271 incident cases and 1,034 mortality cases of 10 respiratory transmitted diseases included in our analyses. Each 5 μg/m^3^ increase in the lag 0–28-day (average of current day and previous 28 days) for wildfire-related PM_2.5_ was associated with a 6.8% (95%CI: 5.0%, 8.7%) increase in the daily incidence rate of respiratory transmitted diseases, which is greater than that of a 1.2% (1.0%, 1.4%) increase associated with the same increase of non-wildfire-related PM_2.5_. A 5 μg/m^3^ increase in wildfire-related PM_2.5_ was associated with a 28.6% (21.0%, 36.8%), 5.2% (2.3%, 8.3%), 12.6% (9.5%, 15.8%), and 13.6% (5.6%, 22.2%) increase in the incidence of seasonal influenza, scarlet fever, rubella, and measles, respectively. Although wildfire-related PM_2.5_ constitutes only 2.7% of the total PM_2.5_, it contributes significantly to respiratory transmitted diseases, accounting for 10.8% of all PM_2.5_-associated cases. In areas where the annual concentration of wildfire-related PM_2.5_ is lower than 1.5 μg/m^3^, the proportion of cases associated with wildfire-related PM_2.5_ reached 29.7%. Study limitations include potential exposure misclassification from using city-average wildfire PM_2.5_ as a proxy for individual exposure and an inability to adjust for some potential confounders.

**Conclusions:**

Short-term exposure to wildfire-related PM_2.5_ was associated with increased incidence of respiratory transmitted diseases, surpassing the impact observed with non-wildfire-related PM_2.5._ This phenomenon is not restricted to regions with high pollutant concentrations; even populations residing in areas with lower concentrations of wildfire-related PM_2.5_ are at an increased risk of these respiratory conditions. Consequently, there emerges a pressing global imperative to confront the escalating challenges presented by climate change and the intensifying menace of wildfires.

## Author summary

**Why was this study done?:**

- The health effects of wildfire smoke on respiratory infections, especially among children and adolescents, remain poorly understood.
- Previous research focused mainly on chronic diseases or COVID-19, with limited data on broader respiratory transmitted diseases.
- As climate change intensifies wildfires globally, understanding their impacts on infectious disease burden is urgently needed.

**What did the researchers do and find?:**

- We analyzed national surveillance data from 501 Chinese cities (2008–2019), covering 6 million respiratory transmitted disease cases in individuals aged 4–24 years.
- Short-term exposure to wildfire-related PM_2.5_ significantly increased the incidence of respiratory transmitted diseases (e.g., influenza, scarlet fever, rubella, measles), with stronger effects than non-wildfire PM_2.5_.
- Even in areas with low wildfire pollution, a large proportion of cases was attributable to wildfire-related PM_2.5_.

**What do these findings mean?:**

- Wildfire-related PM_2.5_ is a potent contributor to respiratory transmitted diseases in children and adolescents, beyond regions with obvious wildfire events.
- Findings highlight the need for global climate action and stronger public health responses during wildfire seasons.
- Main limitations: exposure estimates were based on city-level averages, which may not reflect individual exposure; the study was conducted in China, so generalizability to other settings requires caution.

## Abstract 3

In a retrospective analysis of national surveillance data in China over a 12 year period, Li Chen and colleagues find that short-term exposure to wildfire-related fine particulate matter (PM2.5) was associated with a higher incidence of respiratory transmitted diseases than non-wildfire-related PM2.5.

## Introduction

Wildfires, intensifying in both frequency and intensity due to climate change-induced increases in environmental temperature and the frequency of extreme heat days, have posed a serious threat to human health [1–5]. In contrast to the direct thermal exposure experienced during wildfires, the resultant smoke has the capacity to traverse extensive distances, spanning hundreds to thousands of kilometers, driven by atmospheric wind currents [1]. This far-reaching dispersion extends the impact of wildfires beyond the immediate vicinity, encompassing a significantly larger geographical area and consequently affecting a more substantial demographic [6]. Wildfire smoke is a complex mixture containing particulate, gaseous pollutants [7], and microbes [8], with fine particulate matter (PM_2.5_) being of paramount concern due to its capacity to deeply penetrate the lungs and reach the alveoli, thus posing a severe threat to the human respiratory system [9,10]. Relative to PM_2.5_ originating from other sources, wildfire-related PM_2.5_, characterized by heightened toxicity—a consequence of its distinct chemical composition and reduced particle size—heightens the propensity for inducing respiratory diseases and other health complications [6,11].

Recent estimations indicate that wildfire-related PM_2.5_ exposure is responsible for approximately 0.62% of all-cause mortality in 749 communities across the world each year [12]. Beyond the increased risk of mortality, short-term acute exposure to wildfire smoke significantly escalates the risk of respiratory and cardiovascular diseases [6,13]. Children and adolescents can be more susceptible to environmental hazards than the general adult population. The health impacts of wildfire-related PM_2.5_ are more pronounced in children and adolescents, affecting a spectrum of health outcomes including respiratory illnesses, birth outcomes, ocular diseases, skin inflammation, cephalalgia, and a decrease in physical activity levels [14]. The vulnerability of this demographic is further compounded by the underdevelopment of their respiratory and immune systems, rendering them more susceptible to the deleterious effects of wildfire-related PM_2.5_.

The association between exposure to wildfire-related PM_2.5_ and infectious diseases remains an under-researched area. Although preliminary studies suggest a potential increase in influenza cases related to wildfire-related PM_2.5_ [15], there is a scarcity of comprehensive research in this field. Additionally, research targeting COVID-19 has found that wildfires amplified the effect of short-term exposure to PM_2.5_ on COVID-19 cases and deaths [16]. Furthermore, wildfire smoke contains both PM_2.5_ and microbes [8], with early studies suggesting microbes may act as potential infectious agents [8]. Exposure to wildfire-related PM_2.5_ also increases exposure to microbes. Therefore, the biological plausibility [17,18] and simultaneous exposure to microbes and wildfire-related PM_2.5_ [8] lead to a research hypothesis that short-term exposure to wildfire-related PM_2.5_ will increase the incidence of respiratory transmitted diseases.

To comprehensively understand the impact of wildfire smoke on the incidence and mortality risks of respiratory transmitted diseases in children and adolescents, we untilized a large-scale China national surveillance among children and adolescents aged 4–24 years during 2008–2019 to thoroughly assess the relationship between short-term exposure to wildfire-related PM_2.5_ and non-wildfire-related PM_2.5_ and the incidence and mortality risk of respiratory transmitted diseases.

## Methods

### Health outcome data

Data pertaining to incident cases and death cases among children and adolescents aged 4–24 years, spanning from 2008 to 2019, were procured from the China Information System for Disease Control and Prevention (CISDCP) [19]. The CISDCP is a nationwide, real-time, internet-based surveillance platform managed by the China CDC. All notifiable infectious diseases are required to be diagnosed according to national diagnostic guidelines issued by the National Health Commission and reported by certified clinicians within 24 hours of confirmation. Reported cases undergo multi-tiered verification at local, provincial, and national CDC levels to ensure diagnostic accuracy and data integrity [20]. Routine audits and quality assurance procedures make consistency across regions. This dataset encompassed comprehensive details on each case, including their place of residence, age, sex, as well as dates of onset of symptoms, diagnosis, and clinical outcomes, inclusive of death where applicable. The data specifically focused on 10 notified distinct respiratory transmitted diseases: seasonal influenza, mumps, tuberculosis, scarlet fever, rubella, pertussis, measles, meningococcal meningitis, leprosy, and diphtheria. A summary of the diagnostic criteria is provided in S1 Text. In this study, incidence was defined as the daily number of newly diagnosed cases—capturing both outpatient and inpatient encounters—reported to the CISDCP in accordance with national surveillance protocols. Each report reflected a clinician-confirmed diagnosis following standardized national criteria. Recurrent infections were considered distinct events if they occurred beyond the same disease episode duration and were reported as new cases. Multiple encounters within a single disease episode were recorded as a single event. Mortality was defined as cause-specific death attributed to the diagnosed infectious disease, as recorded in the CISDCP. All deaths were certified by licensed clinicians and reported through the national notifiable disease reporting system. The cause of death corresponds to the primary diagnosis that initiated the reporting, in accordance with national surveillance protocols. The rationale behind selecting the age range of 4–24 years was twofold: it commenced from the typical kindergarten enrollment age of 4 years, thereby encapsulating the educational monitoring phase, and extended to the upper boundary of 24 years, a threshold consistent with the United Nations’ definition of “youth” (15–24 years old) [21]. This demarcation was strategically chosen to ensure a comprehensive inclusion of the educational age range within our study—based on practical considerations of data accessibility and nationwide comparability—thereby enhancing the relevance of our findings to the specific context of China and potentially extending its applicability to analogous nations. Cases emanating from Hong Kong, Macao, Taiwan China, and other nations lacking authenticated records were excluded from our study.

The research encompassed an aggregate of 501 Chinese prefecture-level cities (S1 Fig), exceeding the 333 officially designated prefecture-level divisions. This broader definition incorporated urban districts of centrally administered municipalities (Beijing, Shanghai, Tianjin, and Chongqing), provincial sub-prefecture units (such as special economic zones), and county-level cities under direct provincial administration. These administrative units were treated as distinct cities to match the spatial resolution of both the disease surveillance data and the population-weighted exposure estimates. This was contingent upon the accessibility of incidence and mortality data pertaining to legally notified infectious diseases. Data on daily incident and mortality counts, encompassing respiratory-related cases, were aggregated across the 501 Chinese prefecture-level regions. Ethical approval was not required for this analysis of anonymous data.

### Data sources and exposure assessment

As detailed previously [22], we estimated global daily landscape fire-sourced outdoor fine particulate matter (PM_2.5_) at 0.25°×0.25° spatial resolution (about 28 km × 28 km at the equator) during 2000–2019 using a combination of the GEOS-Chem chemical transport model and machine learning calibration. Briefly, wildfire-specific PM_2.5_ was estimated as the difference between two GEOS-Chem simulations—one including wildfire emissions and one excluding them. Total PM_2.5_ outputs from GEOS-Chem (original resolution 2.0° × 2.5°) were downscaled to 0.25° × 0.25° resolution using inverse distance weighting to match meteorological and auxiliary data grids. A random forest machine learning model was then trained to calibrate GEOS-Chem total PM_2.5_ estimates against ground-based daily PM_2.5_ measurements, using meteorological variables (temperature, relative humidity (RH), wind speed, precipitation, pressure, UV radiation) and spatiotemporal factors (longitude, latitude, day of year, day of week, month, year). The calibrated wildfire-specific PM_2.5_ was obtained by multiplying the calibrated total PM_2.5_ by the GEOS-Chem wildfire-to-total PM_2.5_ ratio in each grid cell. Model validation was conducted using 10 well-documented large wildfire events across the USA, Australia, Portugal, Chile, and South Africa, demonstrating strong agreement between predicted wildfire PM_2.5_ and observed PM_2.5_ peaks and temporal trends, particularly during fire episodes. Non-wildfire PM_2.5_ was calculated as the difference between calibrated total PM_2.5_ and wildfire-specific PM_2.5_. We derived hourly meteorological data at 0.25° × 0.25° spatial resolution from the fifth-generation European Centre for Medium-Range Weather Forecasts reanalysis [23]. Hourly records were used to calculate daily metrological parameters according to local time zone of each grid. These daily metrological parameters included daily mean 2 m (i.e., at 2 m above the surface of the earth) ambient temperature (*T*_mean_ was calculated from 24 hourly records of 2 m ambient temperature), daily mean 2 m dew point temperature (*T*_dew_mean_), daily total precipitation, daily mean surface air pressure. Daily mean RH was calculated from *T*_mean_ and *T*_dew_mean_ using the humidity R package [24]. We collected annual population counts data at 30 arc seconds (about 1 km^2^) spatial resolution across the globe during 2000–2019 from the WorldPop project [25]. We aggregated the gridded population counts to 0.25° × 0.25° spatial resolution to match the air pollution data. For each day, city-level daily PM_2.5_ and weather variables were calculated by averaging the daily exposure of all 0.25° × 0.25° grids within the city boundary weighted by population size of each grid.

## Statistical analysis

### Assessing the wildfire-related PM_2.5_-incidence/mortality association

The time-stratified case-crossover design was employed to explore the potential association between short-term wildfire-related PM_2.5_ exposure and incidence and mortality of all respiratory transmitted diseases and specific respiratory diseases. This design involved comparing the daily average wildfire-related PM_2.5_ levels during the risk period with corresponding control periods within the same city [26–28]. Control periods were meticulously identified, ensuring alignment with the day of the week in the identical calendar month and year corresponding to the observed incidence and mortality cases. Each case was systematically paired with three to four control periods, facilitating a comprehensive analysis that accounted for both time-varying elements such as temporal trends and weekdays, and time-invariant factors including individual demographics (e.g., sex, age, lifestyles), disease types, and the socio-environmental fabric of the respective cities.

We utilized a conditional logistic regression model to examine the relationship between wildfire-related PM_2.5_ concentration and incidence/mortality rates. Wildfire-related PM_2.5_ concentration served as the independent variable, while the dependent variable was a binary indicator (1 = case, 0 = control) distinguishing observations as either cases or controls. To examine the lag structure of the association between wildfire-related PM_2.5_ exposure and respiratory transmitted diseases, a distributed lag nonlinear model (DLNM) was used. Preliminary analysis indicated that this association persisted for up to 28 days. We employed both linear and non-linear (B-spline with 3 degrees of freedom, df = 3) function in the exposure–response dimension and a natural cubic spline with three degrees of freedom for the lag–response relationship over a period of 0–28 days. This lag window aligns with the incubation periods of most included diseases, such as seasonal influenza (1–7 days), mumps (2–4 weeks), scarlet fever (1–12 days), rubella (14–21 days), measles (7–14 days), pertussis (7–14 days), and meningococcal meningitis (2–10 days), supporting its biological plausibility.

The placement of knots in the lag–response dimension was determined by equally spaced values on a logarithmic scale, as per the “logknots” function in the “dlnm” package. The number of knots was set to the degrees of freedom minus one. In the DLNM framework, odds ratio (ORs) were estimated using a linear exposure–response function, relative to a reference value of 0 μg/m³ wildfire-related PM_2.5_, which served as the centering value in the cross-prediction function. Cumulative ORs were computed across the lag period with this same reference. The model also accounted for mean temperature, daily RH, and daily precipitation (all at lag 0 days) by incorporating them with a natural cubic spline of three degrees of freedom, to control for their potential nonlinear impacts. Additionally, total PM_2.5_ concentrations on lag 0 days were adjusted in the model.

The model is formalized as:


$$\begin{array}{cc} o & git\left(P\left(\text{case}=\text{1}|\text{Wildfire}\;{PM}_{\text{2}.\text{5}},\;\text{temp},\;\text{RH},\;\text{precipitation},\text{total}\_{PM}_{\text{2}.\text{5}}\right)\right)=\alpha_{\text{stratumij}}\\ +\; & \text{cb}\left(\text{Wildfire}\;{PM}_{\text{2}.\text{5}}\right)+\text{ns}(\text{tmean},\;\text{df}=\text{3})+\text{ns}(\text{RH},\;\text{df}=\text{3})+\text{ns}(\text{precipitation},\;\text{df}=\text{3})\\ +\; & \beta \times \;\text{total}\_{PM}_{\text{2}.\text{5}} \end{array}$$

Here, $\alpha_{\text{stratumij}}$: stratum-specific intercept, accounting for the matched design in the time-stratified case-crossover analysis. $\text{cb}\left(\text{Wildfire}\;{\text{PM}}_{\text{2}.\text{5}}\right)$: cross-basis function combining a linear exposure–response function and a natural cubic spline (df = 3) for lag–response over a 0–28 day lag period. ns(tmean, df=3), ns(RH, df=3), ns(precipitation, df=3): natural cubic splines controlling for potential non-linear effects of temperature, RH, and precipitation. $\text{total}\_{PM}_{\text{2}.\text{5}}$: daily mean total PM_2.5_ concentration at lag 0.

Our investigation incorporated stratified analyses to assess the impact of wildfire-related PM_2.5_ across diverse demographics and environmental conditions. The stratification criteria included gender (female and male), age categories (4–9, 9–12, 12–15, 15–18, and 18–24 years), the four meteorological seasons (spring, summer, autumn, and winter), and regions categorized by high or low levels of wildfire-related PM_2.5._ Regions with an annual mean wildfire-related PM_2.5_ concentration of 1.5 μg/m^3^ or higher during 2008–2019 were classified as high exposure areas, while those with concentrations below this threshold were classified as low exposure areas. The selection of the 1.5 μg/m^3^ threshold primarily derives from the distributional analysis conducted within the scope of the present study. Specifically, this threshold delineates regions wherein approximately 50% of the area exhibits exposure levels to wildfire-related PM_2.5_ surpassing the aforementioned concentration. We applied separate conditional logistic regression models for each subgroup to derive subgroup-specific ORs estimates, thus facilitating a nuanced understanding of the differential impacts of wildfire-related PM_2.5_ exposure across various population segments and environmental contexts.

Calculating the attributable fraction (AF) of incidence cases due to wildfire-related PM_2.5_

For AF calculations only, the OR obtained from our case-crossover analysis is interpretable as a relative risk (RR), as the control selection scheme based on density sampling leads to control times that represent the average exposure in the study population [29,30]. The RR quantified the likelihood of an incidence or mortality event happening for each 5-unit increase in the daily mean concentration, which aligns with previous studies and ensures comparability across exposure contexts, including during high-exposure wildfire events [31,32]. The RRs were further represented as percent change (PC): PC = (RRs − 1) * 100%.

To determine the annual number of attributable incidence cases/deaths of infectious diseases associated with wildfire-related PM_2.5_, we used the formula to calculate the attributable cases (AC) of day i: ACi =[(exp(β×△x− 1)/exp(β×△x)] ×Ci. Here, *β* represents the log(RR) denoting the cumulative RR over specific lag days associated with a unit increase in wildfire-related PM_2.5_ on each day. Ci represents the city-specific average of incidence cases from the day to the day with specific lag days. Δ*x* represents the differences between the mean daily wildfire PM_2.5_ concentration across the cities and the 0 μg/m^3^. The total number of AC was obtained by summing the ACi values over the study period. Then, we calculated the attributable annual incidence rate by dividing the total AC by the mean population and the number of years covered by the study period specific to each city.

We also applied the same modeling process to PM_2.5_ originating from non-wildfire sources (non-wildfire-related PM_2.5_). We calculated the annual number of attributable incidence cases and deaths from infectious diseases associated with non-wildfire-related PM_2.5_ using 0 μg/m³ as the counterfactual (reference) concentration. Furthermore, we estimated the total number of cases attributable to PM_2.5_ (including non-wildfire-related PM_2.5_ and wildfire-related PM_2.5_) in general and specifically discerned the proportion of these cases that could be uniquely attributed to wildfire-related PM_2.5_ exposure.

### Sensitivity analyses

To test whether 28 days were sufficient to capture the lag effects of wildfire-related PM_2.5_, our study implemented sensitivity analyses that varied the maximum lag for wildfire-related PM_2.5_. These analyses included adjustments to the initial 28-day period, shortening it to 14 days and extending it from 21 to 35 days. Additionally, to test the robustness of our results, we modified the degrees of freedom for meteorological variables to 4, 5, and 6. Furthermore, the meteorological variables at lag 0 were compared with the moving averages of these variables during the 0–28 day lag period.

To mitigate potential exposure misclassification arising from the spatial resolution of the exposure grid, we performed a sensitivity analysis restricted to large cities, defined as those with both a geographic area >500 km^2^ and a population >1 million. To determine the statistical significance of differences in the estimated ORs across these varied sensitivity analyses, we implemented a univariate fixed-effects meta-regression. This technique was crucial in identifying any potential statistical discrepancies among the results. Essentially, our approach entailed constructing models that incorporated effect estimates from different sensitivity analyses alongside their respective standard errors (SEs), correlated with meta-predictors. The meta-regression model then facilitated the assessment of differences in effect estimates between strata using the likelihood ratio test.

We used R software (version 4.2.2) to perform all data analyses. The packages “gnm,” “dlnm,” and “mvmeta” were used to fit conditional logistic regression, distributed lag linear or non-linear model, and meta-regression model, respectively. A two-sided *p* value less than 0.05 was considered to be statistically significant.

## Results

### Descriptive statistics

**Fig 1:**
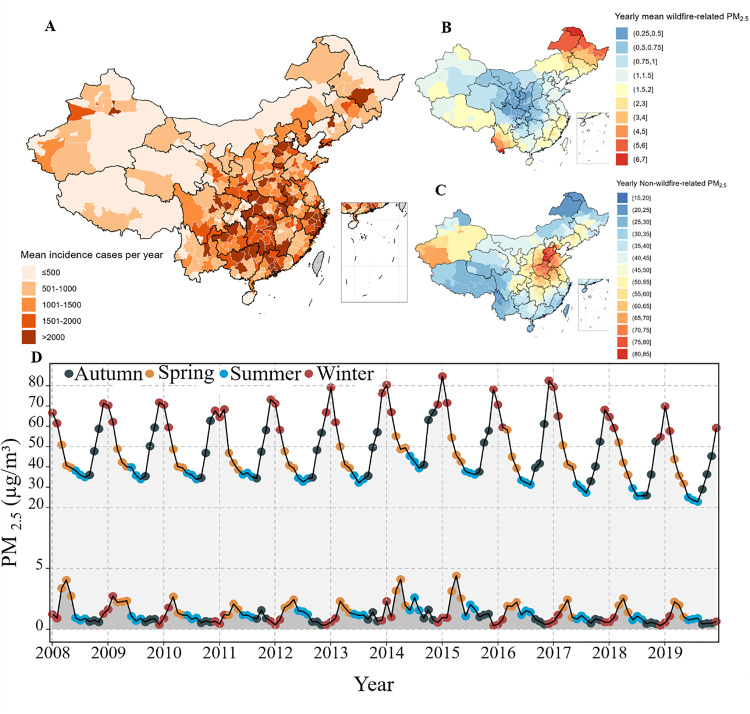
Annual spatial distribution of incidence cases and concentrations of wildfire-related vs. non-wildfire-related PM_2.5_, with temporal trends. Panel **A** presents the mean number of incident cases from 2008 to 2019; Panel **B** shows the population-weighted means of wildfire-related PM_2.5_; Panel **C** displays the population-weighted means of non-wildfire-related PM_2.5_; and Panel **D** illustrates the monthly population-weighted means of both wildfire-related (the lower line) and non-wildfire-related PM_2.5_ (the higher line). PM_2.5_ refers to particulate matter with a diameter of less than 2.5 micrometers. Notes; Spatial boundaries were retrieved from Natural Earth (https://www.naturalearthdata.com/) using the “rnaturalearth” package (https://github.com/ropenscilabs/rnaturalearth).

This study included 6,089,271 incident cases (female, 65.6%; male, 34.6%) and 1,034 death cases (female, 47.1%; male, 52.9%) during 2008–2019. Among these incident cases, seasonal influenza was identified as the predominant infectious disease, with the largest number of cases at 2,255,929 and a median age of 9.6 years, followed by mumps (2,155,598 cases with a median age of 9.8), tuberculosis (1,074,814 cases with a median age of 19.1), scarlet fever (294,059 cases with a median age of 7.3), rubella (256,061 cases with a median age of 13.8), measles (45,278 cases with a median age of 12.0), pertussis (5,722 cases with a median age of 7.6), meningococcal meningitis (1,405 cases with a median age of 13.0), and leprosy (405 cases with a median age of 18.0). Among mortality cases, tuberculosis was the most common cause of death, with 708 cases and a median age of 19 years, followed by seasonal influenza, meningococcal meningitis, measles, mumps, and scarlet fever, each contributing to the overall mortality figures with varying case numbers (Tables 1 and S1 Table). The spatial distribution of the incident cases and death cases is visualized in Figs 1 and S2.

**Table 1:** Descriptive of specific-cause respiratory transmitted diseases counts in China.

	Incidence case						Incidence case					
	Number	Female	Male	Age			Number	Female	Male	Age		
				Min	Median	Max				Min	Median	Max
Overall respiratory transmitted diseases	6,089,271	3,996,589	2,092,682	–	–	–	1,034	487	547	–	–	–
Seasonal influenza	2,255,929	1,252,309	1,003,620	4.9	9.6	23.0	214	134	80	6	17	23.0
Mumps	2,155,598	1,525,348	630,250	4.2	9.8	23.0	3	1	2	6	13.5	22.4
Tuberculosis	1,074,814	762,876	311,938	4.9	19.1	23.0	708	311	397	5.9	19	23
Scarlet fever	294,059	199,347	94,712	5.0	7.3	23.0	3	1	2	6	12	13
Rubella	256,061	209,991	46,070	4.8	13.8	23.0	–	–	–	–	–	–
Measles	45,278	41,613	3,665	5.0	12.0	23.0	15	5	10	6.3	8	22.3
Pertussis	5,722	3,693	2,029	5.3	7.6	22.9	–	–	–	–	–	–
Meningococcal meningitis	1,405	1,132	273	5.7	13.0	23.0	91	35	56	5.7	12	21.8
Leprosy	405	280	125	6.0	18.0	22.8						

In China, the average total PM_2.5_ concentration was measured at 49.6 μg/m^3^, with wildfire-related PM_2.5_ contributing approximately 1.3 μg/m^3^, equating to about 2.7% of the total. Observing the long-term yearly mean distributions, there is a notable divergence in the trends of wildfire-related and non-wildfire-related PM_2.5_ (S3 Fig and S2 Table). The areas most impacted by wildfire-related PM_2.5_ include the northeastern and southwestern regions, along with select coastal zones within China. Conversely, the central regions of China are predominantly affected by higher concentrations of non-wildfire-related PM_2.5_ (Fig 1).

### Associations by different respiratory transmitted diseases

We observed that exposure to wildfire-related PM_2.5_ was associated with a lower incidence of overall respiratory diseases in the first 6 days, and the risk of incidence increased from day 6 to 28. Thereafter, the associations gradually attenuated and became statistically nonsignificant after 28 days (S4 Fig). Consequently, we decided to use the duration of 0 to 28 days to derive the risk estimates. Similarly, for mortality, we utilized the same lag duration to determine the risk estimates.

Every 5 μg/m^3^ increase in the concentrations of wildfire-related PM_2.5_ was associated with a 6.8% increase (95% CI: 5.0%, 8.7%) in the incidence of overall respiratory transmitted diseases. Additionally, this increase in wildfire-related PM_2.5_ was associated with a 28.6% (95% CI: 21.0%, 36.8%), 5.2% (95% CI: 2.3%, 8.3%), 12.6% (95% CI: 9.5%, 15.8%), and 13.6% (95% CI: 5.6%, 22.2%) increase in the incidence of seasonal influenza, scarlet fever, rubella, and measles, respectively.

**Fig 2:**
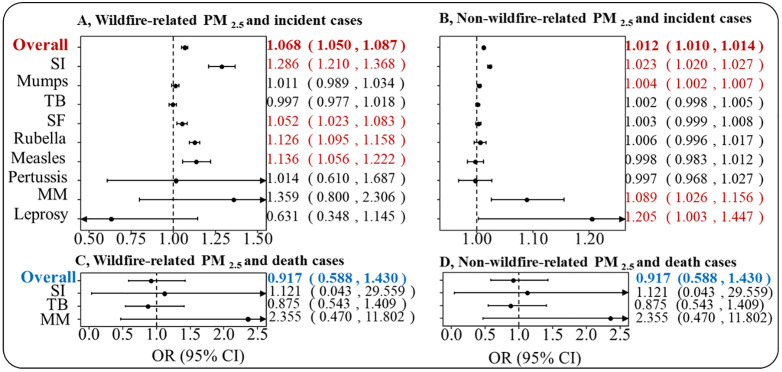
Estimated associations between respiratory infectious cases and death and wildfire-related or non-wildfire-related PM_2.5_ exposure (every 5 μg/m^3^ increase). Notes: Subfigure **A** presents the estimated association between incident cases of respiratory infections and wildfire-related PM_2.5_; Subfigure **B** presents the estimated association between incident cases of respiratory infections and non-wildfire-related PM_2.5_; Subfigure **C** presents the estimated association between death cases of respiratory infections and wildfire-related PM_2.5_; Subfigure **D** presents the estimated association between death cases of respiratory infections and non-wildfire-related PM_2.5_. SI, Seasonal influenza; TB, Tuberculosis; SF, Scarlet fever; MM, Meningococcal meningitis. Models are time-stratified case-crossover (conditional logistic regression) adjusted for daily mean temperature, relative humidity, precipitation, and total PM_2.5_.

Conversely, every 5 μg/m^3^ increase in non-wildfire-related PM_2.5_ was linked to a 1.2% (95% CI: 1.0%, 1.4%) increment in the overall incidence of respiratory transmitted diseases. This increase in non-wildfire-related PM_2.5_ also showed correlations with specific diseases: a 2.3% (95% CI: 2.0%, 2.6%) increase for seasonal influenza, a 0.4% (95% CI: 0.2%, 0.7%) rise for mumps, an 8.9% (95% CI: 2.6%, 15.6%) augmentation for meningococcal meningitis, and a 20.5% (95% CI: 0.3%, 44.0%) escalation for leprosy. Null associations were observed between death cases and exposure to both wildfire-related and non-wildfire-related PM_2.5_ (Fig 2).

**Fig 3:**
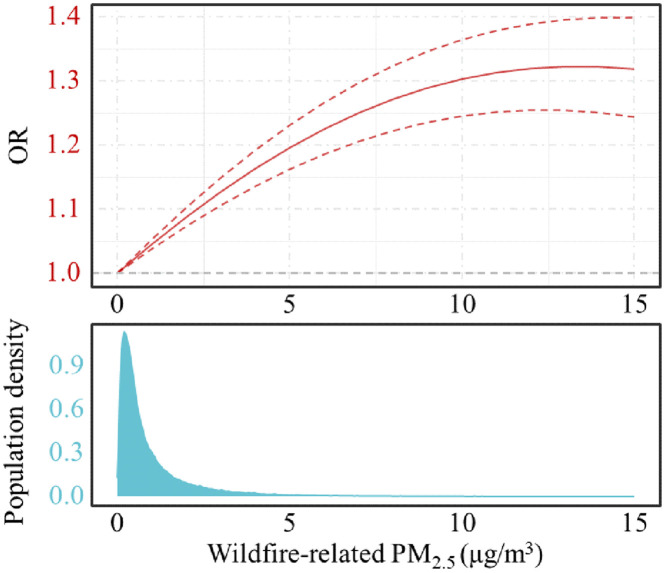
Estimated nonlinear associations between wildfire-related PM_2.5_ and respiratory transmitted diseases. Notes: The figure above illustrates the estimated non-linear association between wildfire-related PM_2.5_ and respiratory transmitted disease. The figure below depicts the population density of wildfire-related PM_2.5_ in the current study. Models are time-stratified case-crossover (conditional logistic regression) adjusted for daily mean temperature, relative humidity, precipitation, and total PM_2.5_.

We evaluated the non-linear relationship between exposure to wildfire-related PM_2.5_ and the incidence of overall respiratory transmitted diseases, as depicted in Fig 3. This evaluation highlighted concentration–response associations, demonstrating positive non-linear curves between daily levels of wildfire-related PM_2.5_ and the incidence of respiratory transmitted diseases. These curves consistently showed an increase in incidence rates with no apparent thresholds. Notably, the slope of these curves varied in response to different concentrations of wildfire-related PM_2.5_ during short-term exposures. Specifically, at wildfire-related PM_2.5_ levels below 3 μg/m^3^, the slope was observed to be steeper, whereas it became more gradual at higher concentration ranges. Given the rarity of wildfire-related PM_2.5_ concentrations >5 μg/m³, estimates in this range reflect sparse data and should be interpreted as indicative of extreme exposure scenarios only.

### Stratified analyses for the estimated associations

We examined whether the estimated association between non-wildfire-related and wildfire-related PM_2.5_ and overall respiratory transmitted diseases differed among subpopulations. Our findings indicated a pronounced gender-based susceptibility, with males exhibiting a higher susceptibility (19.9% [95% CI: 14.8%, 25.2%]) to the adverse effects of wildfire-related PM_2.5_ compared to females (4.7% [95% CI: 3.1%, 6.4%]). Among the different age groups, children and adolescents aged 9–12 years showed the greatest vulnerability to the adverse effects of wildfire-related PM_2.5,_ with an increase of 8.9% (95% CI: 5.9%, 11.9%). Additionally, the age groups of 4–9 years and 12–15 years also demonstrated heightened susceptibility to these effects, with increases of 7.3% (95% CI: 4.8%, 10.0%) and 7.7% (95% CI: 4.5%, 11.0%), respectively (S5 Fig). The stratified analysis for the seasonal influenza, scarlet fever, rubella, and measles shown in S6 Fig.

Our analysis incorporated stratified assessments based on the four meteorological seasons to evaluate the impact of PM_2.5._ The findings indicated that the adverse effects of wildfire-related PM_2.5_ were most pronounced during the autumn season, with the highest OR value of 1.519 (95%CI: 1.431, 1.612), as illustrated in S5A Fig. Notably, this occurred despite the concentration of wildfire-related PM_2.5_ being the lowest among the four seasons (as shown in Fig 1D). In contrast, the impact of non-wildfire-related PM_2.5_ during the autumn and summer seasons displayed similar patterns of adverse effects, as depicted in S5B Fig.

The stratified analysis, differentiated by regions with high or low levels of wildfire-related PM_2.5_, revealed that the adverse effects of wildfire-related PM_2.5_ were more pronounced in regions with low levels of this pollutant, exhibiting an OR of 1.284 (95% CI: 1.239, 1.331). In contrast, the adverse effects of non-wildfire-related PM_2.5_ were greater in regions with high levels of wildfire-related PM_2.5_, with an OR of 1.021 (95% CI: 1.016, 1.027).

### Comparative impacts from wildfire-related and non-wildfire-related PM_2.5_

**Fig 4:**
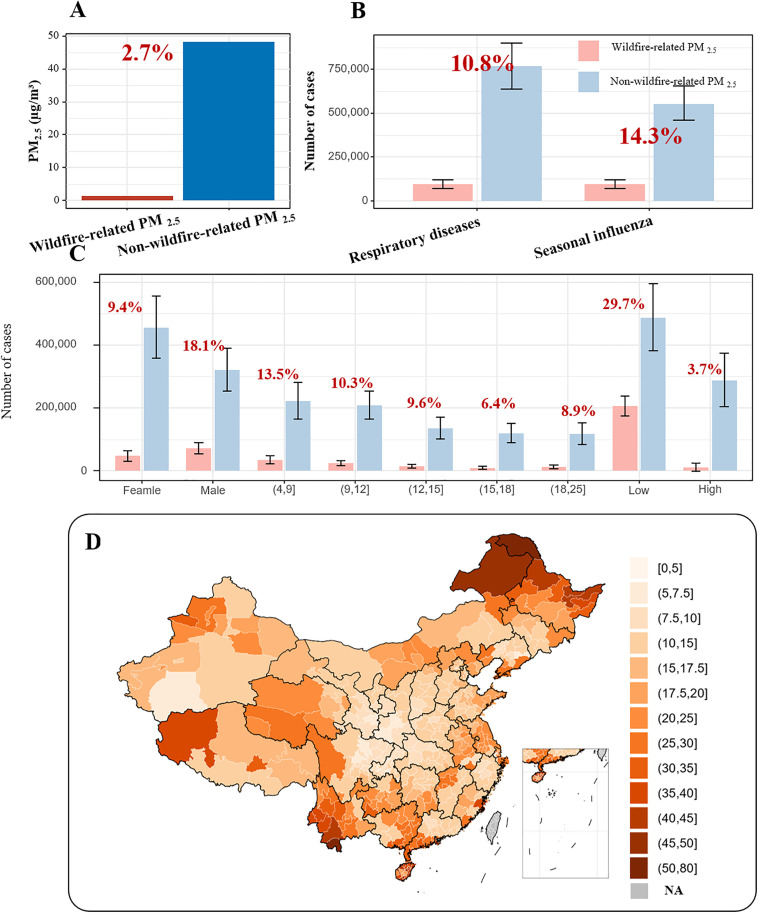
Health impacts of PM_2.5_ from wildfire and non-wildfire sources. Notes: Proportion indicates the contribution of wildfire-related PM_2.5_ to PM_2.5_-linked respiratory transmitted diseases. Subfigure **A** displays the proportion of wildfire-related PM_2.5_ and non-wildfire-related PM_2.5_ in total PM_2.5_. Subfigure **B** illustrates the number of respiratory transmitted diseases and specific diseases (e.g., seasonal influenza), along with the proportion of attributable cases from wildfire-related PM_2.5_, defined as [attributable to wildfire-related PM_2.5_] ÷ ([attributable to wildfire-related PM_2.5_] + [attributable to non-wildfire PM_2.5_]). Subfigure **C** presents the number of respiratory transmitted diseases and their proportions attributed to wildfire-related PM_2.5_ and non-wildfire-related PM_2.5_, categorized by gender groups, age groups, and high or low exposure areas. Subfigure **D** shows the proportion of respiratory transmitted diseases attributed to wildfire-related PM_2.5_ by provinces. “NA” denotes provinces/diseases with unavailable data. Spatial boundaries were retrieved from Natural Earth (https://www.naturalearthdata.com/) using the “rnaturalearth” package (https://github.com/ropenscilabs/rnaturalearth).

Fig 4 illustrated the comparative analysis of the impacts attributed to wildfire-related PM_2.5_ versus non-wildfire-related PM_2.5_. It was discerned that wildfire-related PM_2.5_ bears a greater significance in public health, despite the fact that high-concentration exposure to wildfire-related PM_2.5_ is less common compared to non-wildfire-related PM_2.5_ (as indicated in Fig 1B and 1C). The analysis underscores that while wildfire-related PM_2.5_ constitutes only 2.7% of the total PM_2.5_, it contributes significantly to respiratory transmitted diseases, accounting for 10.8% of all PM_2.5_-associated cases. Particularly for seasonal influenza, the proportion of cases associated with wildfire-related PM_2.5_ reaches 14.3% of the total PM_2.5_-associated respiratory transmitted diseases. Furthermore, the incidence of cases associated with wildfire-related PM_2.5_ varies across different genders and age groups. In regions with lower levels of wildfire-related PM_2.5_ 29.7%), the proportion of associated cases is greater than that in regions with higher levels (3.7%). The distribution of the proportion of cases related to wildfire-related PM_2.5_ was calculated based on the regional variations in wildfire-related PM_2.5_ levels. The distribution of the proportion based on the national estimated association shown in S7 Fig.

The robustness of the findings was demonstrated through sensitivity analyses, which involved changing the maximum lag time for wildfire-related PM_2.5_ to 27, 29, 30, and 31 days. We also adjusted the degrees of freedom for meteorological variables to 4, 5, and 6, and compared meteorological variables at lag 0 with their averages over a 0–28 day period. These analyses, detailed in S3 Table, ensured the reliability of our findings and for assessing the potential variability in our results under different analytical conditions.

## Discussion

### Principal findings

Leveraging a comprehensive nationwide surveillance dataset spanning 12 years, our study established a significant correlation between exposure to wildfire-related PM_2.5_ and an elevated incidence of respiratory transmitted diseases. However, no significant association with mortality was observed. We found that exposure to wildfire-related PM_2.5_ was significantly associated with increased incidence in respiratory transmitted diseases, and specific diseases including seasonal influenza, scarlet fever, rubella, and measles at a national level, but the associations varied across different demographics, seasons, and geographical regions.

By comparison, we found that wildfire-related PM_2.5_ had stronger effects on the respiratory transmitted diseases than other source PM_2.5_, with a 6.8% increase in incidence of overall infectious diseases for wildfire-related PM_2.5_ and a 1.2% increase in incidence of overall infectious diseases for non-wildfire-related PM_2.5_. And compared with non-wildfire-related PM_2.5_, wildfire-related PM_2.5_ only accounted 2.7% of total PM_2.5_, while accounting for 14.7% of all PM_2.5_-associated cases. These findings likely reflect both the episodic nature and higher toxicity of wildfire-related PM_2.5_, consistent with prior evidence [12,32,33]. Wildfire-related PM_2.5_ differs in chemical composition from urban background PM_2.5_; it contains higher fractions of organic carbon, polycyclic aromatic hydrocarbons, black carbon, and reactive oxygen species, which have been shown to induce stronger oxidative stress, inflammation, and immune dysfunction [34,35]. Previous studies have demonstrated that wildfire PM_2.5_ is associated with disproportionately higher health risks per unit exposure compared with other PM_2.5_ sources, including greater impacts on respiratory and cardiovascular outcomes [34–36].

Previous studies have also reported the deleterious effects of PM_2.5_’s on various respiratory diseases, including COPD [37,38], asthma [39,40], lung cancer [41], idiopathic pulmonary fibrosis [42,43], acute respiratory infections [44,45], bronchiectasis [46], and tuberculosis [47,48]. However, most of this research has focused on chronic non-communicable conditions. Our study expands this evidence base by highlighting the impact of PM_2.5_—particularly from wildfires—on respiratory infections. Notably, recent studies have shown that wildfire-related PM_2.5_ exacerbates COVID-19 incidence and mortality [16,49]. Given that both COVID-19 and influenza are icosahedral respiratory viruses sharing similar transmission routes and immune vulnerabilities, air pollution may amplify risks through shared mechanisms. Evidence from wildfire-prone regions such as California and Brazil supports this association [50–53]. Our findings align with this literature and suggest that even short-term wildfire PM_2.5_ exposure can impair host defenses and promote viral spread, underscoring the need for coordinated air quality and epidemic control strategies during wildfire seasons.

We also assessed the nonlinear exposure–response between the wildfire-related PM_2.5_ and incidence of respiratory transmitted diseases, which suggests that there is no safe threshold of the wildfire-related PM_2.5_ exposure. Although the national analysis indicates a non-linear association between the concentration of wildfire-related PM_2.5_ and the incidence of respiratory transmitted diseases, it is noteworthy that majority of the sample are concentrated within the 0–5 μg/m^3^ range of wildfire-related PM_2.5_ concentration. Within this concentration range, the association between wildfire-related PM_2.5_ and the incidence of respiratory transmitted diseases tends to be linear. Additionally, as the concentration of wildfire-related PM_2.5_ increases, the confidence interval correspondingly expands. Compared to previous studies on the non-linear association of the total PM_2.5_, our study finds that the effects are more pronounced at lower concentrations, with the slope becoming gentler at higher concentrations, similar to previous findings [54–58]. The difference lies in the fact that a large portion of the population is actually exposed to lower concentrations of wildfire-related PM_2.5_.

Wildfire-related PM_2.5_ may promote the onset of respiratory infections through several biological mechanisms. Wildfire smoke, represented by wildfire-related PM_2.5_, has a more complex composition than non-wildfire PM_2.5_, including microbes [8]. Due to the lack of assessment of wildfire-related microbes, a direct evaluation of the association between microbes and infectious diseases cannot be conducted. This suggests that the influence of wildfire smoke on infectious diseases is more complex than PM_2.5_ alone, involving the interaction between microbes in wildfire smoke and wildfire PM_2.5_. One supporting piece of evidence is the increased risk of fungal infections following exposure to wildfire smoke [59]. In the areas with lower level of wildfire-related PM_2.5_ concentrations, up to 42.6% of respiratory transmitted disease cases associated with PM_2.5_ can be specifically attributed to wildfire exposure. This may be due to the smaller particle size of particulates from wildfires compared to those from urban sources. After long-distance transport, these smaller particles may possess greater oxidative capacity [60,61]. Furthermore, despite lower concentrations after long-distance transport and particulate settling, the remaining particles could potentially pose a greater health hazard. Such properties may contribute to greater biological activity and increased incidence of infectious diseases, even at lower ambient concentrations. Currently, research on the mechanisms by which PM_2.5_ impacts the incidence of infectious diseases is limited. Nevertheless, we hypothesize several potential pathways. First, PM_2.5_ may increase the risk of infection by impairing the respiratory tract and lung barrier function, thereby facilitating pathogen invasion and increasing the likelihood of disease onset [62,63]. Second, PM_2.5_ could exacerbate infection-related symptoms by inducing oxidative stress and inflammatory responses, disrupting the immune system’s equilibrium, and thereby increasing the risk and severity of infectious diseases [62–64]. It is important to note that current research on these mechanisms is relatively limited, necessitating further comprehensive investigations to fully understand the impact of PM_2.5_ on infectious diseases.

Multiple studies indicates that climate change is poised to escalate both frequency and intensity of wildfires in the future [1,3,65,66]. Prior investigations have revealed that billions of people globally are exposed to substantial wildfire air pollution, especially in hotspot and underdeveloped regions [22]. However, our research extends these findings, showing that even populations in areas not directly afflicted by intense wildfire pollution experience substantial effects, underscoring the far-reaching, global ramifications of climate change. This highlights the necessity for extensive international cooperation to curtail global temperature increases and mitigate wildfire incidents effectively [65]. Earlier research supports this approach, indicating that maintaining the global average temperature increase within 2.0 or 1.5 °C above pre-industrial levels could avert 60% or 80% of the projected escalation in wildfire exposure, respectively [6,65].

### Strengths and limitations

The study leveraged a comprehensive national dataset, notable for its substantial sample size and extensive coverage across geographic regions. This dataset, comprising data from 6 million cases involving children and adolescents, provided a unique opportunity for a systematic and consistent evaluation of associations. Our study encompassed a wide range of wildfire-related pollution levels, allowing us to provide more representative estimates of the effects of short-term wildfire-related PM_2.5_ exposure on respiratory transmitted diseases. Several limitations of this study should be noted.

There is a potential for exposure measurement error due to the use of city average values as proxies for individual exposure levels. The average exposure calculations for larger geographic areas may not precisely reflect the individual exposure levels to wildfire-related air pollution, which can vary significantly within a city. Additionally, the 0.25° × 0.25° (~28 km) grid resolution of the exposure dataset may not capture fine-scale spatial heterogeneity, particularly in small or topographically complex cities. Additionally, the inability to access the specific address data of children and adolescents for privacy reasons might have introduced non-differential error, typically leading to an attenuation of effect estimates [67]. The average exposure calculations for larger geographic areas may not precisely reflect the individual exposure levels to wildfire-related air pollution, which can vary significantly within a city. Despite the CISDCP encompassing over 95% of health facilities, there remains a possibility of omission or underreporting of some infectious and mortality cases. Such underreporting could introduce errors in the outcome measurement, potentially biasing the associations between wildfire-related PM_2.5_ and respiratory transmitted diseases towards null, under the assumption that these inaccuracies are not related to wildfire-related PM_2.5_ exposure. The data was sourced from China, implying that its conclusions might not be entirely applicable to other countries or regions with different wildfire patterns and air pollution components. However, to our knowledge, this study is the first to encompass all regions of China and include over 330 million children and adolescents aged 4–24 years, providing large-scale evidence for the association between wildfire-related PM_2.5_ and respiratory transmitted diseases. Although geographical and environmental differences may limit the generalizability of the study results, the large scale and comprehensiveness of this research make it a significant reference for understanding the impact of wildfire PM_2.5_ on human health. This study focused on individuals aged 4–24 years, aligning with the formal education age range in China. However, several respiratory transmitted diseases—notably measles, pertussis, meningococcal meningitis, and diphtheria—commonly affect children under 5 years of age. Excluding children younger than three may result in an underestimation of the real disease burden and limit the generalisability of our findings to early childhood. Further research is needed to evaluate the health impacts of wildfire-related PM_2.5_ exposure in younger children. Additionally, the exposure window used in this study (lag 0–28 days) is more appropriate for acute infections with short incubation periods. Interpretations of associations for chronic infectious diseases, such as tuberculosis or leprosy, should therefore be made with caution. Finally, the limited number of mortality events in our dataset substantially constrained statistical power for detecting associations with mortality outcomes; as such, these results should be interpreted with caution and warrant further investigation in larger datasets. Although a linear exposure–response function was applied, the +5 µg/m³ increment may reflect an extreme value in some regions, potentially introducing instability in lag-specific estimates, particularly in early lags and low-exposure settings.

Employing an extensive national dataset and established statistical methodologies, our study identified a robust association between short-term exposure to wildfire-related PM_2.5_ and an increased incidence of respiratory transmitted diseases, with greater impact compared with non-wildfire-related PM_2.5_. The high proportion of respiratory transmitted disease cases associated with wildfire-related PM_2.5_, out of all cases associated with total PM_2.5_ in areas with lower concentrations of wildfire-related PM_2.5_, confirms the extensive impact of wildfires on human health. Compared to the direct effects in high-concentration areas, populations in low-concentration areas also face a higher risk of respiratory transmitted diseases.

## Supporting information

S1 Text Summary of diagnostic criteria for 10 notifiable infectious diseases. (DOCX)

S1 Table The descriptive of specific-cause respiratorily transmitted infectious diseases counts in China. (XLSX)

S2 Table Descriptive of daily wildfire-related PM25 and O3 concentration. (XLSX)

S3 Table Results of sensitive analysis. (XLSX)

S1 Fig The locations of the 501 Chinese prefecture-level cities included in the present study. Notes: Spatial boundaries were retrieved from Natural Earth (https://www.naturalearthdata.com/) using the “rnaturalearth” package (https://github.com/ropenscilabs/rnaturalearth).
(TIF)

S2 Fig Distribution of death cases. Notes:Spatial boundaries were retrieved from Natural Earth (https://www.naturalearthdata.com/) using the “rnaturalearth” package (https://github.com/ropenscilabs/rnaturalearth).
(TIF)

S3 Fig Annual spatial distribution of incidence cases and concentrations of wildfire-related with temporal trends. (TIF)

S4 Fig Association between wildfire-related PM_2.5_ exposure (per 5 μg/m^3^ increase) and incidence and mortality of respiratory transmitted diseases across lag 0–28 days, using a linear exposure-response function. (TIF)

S5 Fig Estimated associations between respiratory infectious cases and wildfire-related or non-wildfire-related PM_2.5_ exposure by subpopulation. Note: Low refers to areas with yearly wildfire-related PM_2.5_ concentrations <1.5 μg/m3; High denotes areas with yearly wildfire-related PM_2.5_ concentrations ≥1.5 μg/m^3^. Subfigure A presents the estimated association between respiratory transmitted disease and wildfire-related PM_2.5_ by subgroups, while Subfigure B presents the estimated association between respiratory transmitted diseases and non-wildfire-related PM_2.5_ by subgroups.
(TIF)

S6 Fig Results of subgroup analysis (TIF)

S7 Fig Proportion that contributed to Wildfire-related PM_2.5_ of PM_2.5_-linked respiratory transmitted diseases based on the national estimated association. Notes; Spatial boundaries were retrieved from Natural Earth (https://www.naturalearthdata.com/) using the “rnaturalearth” package (https://github.com/ropenscilabs/rnaturalearth).
(TIF)

S4 Table Respiratory infectious disease cases attributable to wildfire-related PM_2.5_ by province, China. (XLSX)

## Abbreviations

AC: attributable cases
AF: attributable fraction
CISDCP: China Information System for Disease Control and Prevention
DLNM: distributed lag nonlinear model
ORs: odds ratio
PC: percent change
PM_2.5_: particulate matter
RH: relative humidity
RR: relative risk
