# Supplementary material for: Wildfire-related PM2.5 and respiratory transmitted disease among Chinese children and adolescents from 2008 to 2019: A retrospective study
Source: PLoS Med. 2025 Dec 5;22(12):e1004613. doi: 10.1371/journal.pmed.1004613 (PMC12680207; doi:10.1371/journal.pmed.1004613)
Supplement: S1 Text — (DOCX) [file pmed.1004613.s001.docx]

**S1_text:** **Summary of diagnostic criteria for 10 notifiable infectious diseases**

**Seasonal Influenza**

Diagnostic Standard: WS 285-2008 (National Health Industry Standard of China)

[url:http://wsbz.nhc.gov.cn/wsbzw/upload/StandardLibrary/5e7e7e77556042a294cca10cfcf1e526.pdf]

**1. Clinical symptoms:** Related clinical symptoms (omitted).

**2. Contact history:** In local epidemic seasons (such as winter and spring in north China, winter, spring and summer in south China), a large number of patients with upper respiratory tract infection occur in one unit or region, or the number of patients with upper respiratory tract infection increases significantly in hospital outpatient service and emergency department.

**3. Laboratory Tests:**

- 1. Isolation and identification of influenza viruses from respiratory tract specimens of patients

3.2 Antibody titers against influenza virus in the convalescent serum were 4 times higher than those in the acute phase.

3.3Influenza virus specific nucleic acid tests are positive or specific antigens are detected in the respiratory tract of the patient.

3.4After the virus is proliferated by sensitive cells, the influenza virus specific nucleic acid is tested positive or the specific antigen is detected.

**4. Case definition principle**

Confirmation of influenza cases requires laboratory testing confirmation.

**Mumps**

Diagnostic Standard: WS 270-2007 (National Health Industry Standard of China)

[url:http://wsbz.nhc.gov.cn/wsbzw/upload/StandardLibrary/b8b45a3dbfb0416eb113e3d6718d0e8c.pdf]

**1. Clinical symptoms：**Related clinical symptoms (omitted).

**2. Contact history:** A history of contact with a mumps patient during 14 to 28 days before onset or a local mumps epidemic.

**3. Laboratory Tests:**

3.1 Leukocyte count and urine routine examination are generally normal, but the leukocyte of the person that has testicular inflammation can heighten.

3.2Serum and urinary amylase increased in 90% of patients at early onset. In patients without parotid enlargement, serum and urinary amylase may also be elevated.

3.3 Viral meningitis with cerebrospinal fluid changes can occur in about half of patients.

3.4 No live attenuated mumps vaccine was inoculated within 1 month. Mumps virus specific IgM antibody was detected in serum.

3.5 The titer of mumps virus IgG antibody was increased by 4 times or more (including antibody positive transformation) between

convalescence and acute phase (2-4 weeks apart).

3.6 Mumps virus was isolated from saliva, urine, cerebrospinal fluid and other body fluids.

**4. Case definition principle**

Diagnosis is based on an epidemiological history, acute enlargement of the parotid gland and/or other salivary glands, except for

parotid enlargement from other causes. Laboratory specific tests are required for confirmed cases.

**5. Source of diagnostic criteria,** detailed testing methods, instruments, procedures, etc:

“WS 270-2007” (Health Industry Standard of China)

<http://wsbz.nhc.gov.cn/wsbzw/upload/StandardLibrary/b8b45a3dbfb0416eb113e3d6718d0e8c.pdf>

**Tuberculosis (TB)**

**1. Clinical symptoms**：Related clinical symptoms (omitted).

**2. Epidemiological history:**

Patients had history of contact with TB patient.

**3. Chest imaging examination**

3.1 Primary pulmonary tuberculosis

Primary pulmonary tuberculosis is characterized by primary pulmonary lesion and intrathoracic lymph node enlargement, or simple intrathoracic lymph node enlargement. Primary pulmonary tuberculosis in children may also present as cavitation, caseous pneumonia, and bronchial tuberculosis due to broncholymphatic fistula.

3.2 Hematogenous disseminated pulmonary tuberculosis

Acute disseminated hematogenous pulmonary tuberculosis is characterized by uniformly distributed miliary shadows of uniform size and density in both lungs. Diffuse focus of subacute or chronic disseminated pulmonary tuberculosis, distributed in the upper middle of both lungs, with different size and density, may have fusion. Acute disseminated hematogenous pulmonary tuberculosis in children is sometimes only presented as a ground glass shadow, and the exudation around the miliary lesions in infants is obvious, with blurred edges and easy fusion.

3.3 Secondary pulmonary tuberculosis

The chest images of secondary pulmonary tuberculosis showed various manifestations. In light cases, it was mainly manifested as plaques, nodules and cord shadow, or tuberculosis tumor or isolated cavity. The severe cases can be manifested as lobular infiltration, caseous pneumonia, multiple cavitation formation and bronchial diffusion, etc. Repeatedly in delay progress can appear lung damage, damage to the lung tissue volume smaller, its multiple thick wall hollow fibre, secondary bronchiectasis, or accompanied by multiple calcification, adjacent to the lung and mediastinum structure retraction of the displacement of thoracic collapse, pleural thickening adhesions on the right, compensatory emphysema and other lung tissue of old and new mixed

endobronchial spread of lesions, etc.

3.4 Tracheal and bronchial tuberculosis

Tracheal and bronchial tuberculosis is mainly manifested as irregular thickening of the trachea or bronchial wall, stenosis or obstruction of the lumen, and secondary atelectasis or consolidation, bronchiectasis and other bronchodiffusion lesions in the distal lung tissues of the bronchial stenosis.

3.5 Tuberculous pleurisy

Tuberculous pleurisy is divided into dry pleurisy and exudative pleurisy. Dry pleurisy is an early inflammatory reaction of the pleura, usually without obvious imaging manifestations. Effusion sex pleurisy basically expresses pleural effusion, and pleural effusion can show for a few or in a great deal of free effusion, or the limitation that exists in pleural any place accumulates fluid, absorb slowly person often amalgamative pleural thicken adhesion, also can evolve pleural tuberculous tumour and empyema to wait.

**4. Laboratory testing**

4.1 Bacteriological examination

The testing method is shown in URL below. The results are as follows:

A) Positive smear microscopy.

B) Mycobacterium culture was positive, and the species was identified as mycobacterium tuberculosis complex group.

4.2 Molecular biology examination

Mycobacterium tuberculosis tested positive for nucleic acid.

4.3 pathological examination of tuberculosis

The histological changes of tuberculosis are shown in URL below.

4.4 Immunological examination

4.4.1 Tuberculin skin test, moderately positive or strongly positive (see URL below).

4.4.2 Positive γ gamma interferon release test.

4.4.3 Mycobacterium tuberculosis antibody positive.

**5. Bronchoscopy**

Bronchoscopy can be used to directly observe trachea and bronchial lesions, as well as suction of secretions, brush examination and biopsy.

**6. Diagnostic principles**

The diagnosis of tuberculosis is mainly based on the examination of etiology (including bacteriology and molecular biology), combined with the epidemiological history, clinical manifestations, chest imaging, relevant auxiliary examination and differential diagnosis, etc. The diagnosis was based on the results of etiology and pathology. The diagnosis of children's tuberculosis, in addition to sputum pathogen examination, but also should be paid attention to gastric

pathogen examination.

**7. Confirmed cases:**

7.1 Sputum smear positive pulmonary tuberculosis diagnosis

If one of the following items is met:

A) The acid-fast bacillus test of 2 item sputum specimens meets 4.1.a item;

B) One sputum specimen with acid-fast bacilli test meets 4.1.a item and meets any of 3 item;

C) One sputum specimen was found to be in line with 4.1.a item and one sputum specimen was found to be in line with 4.1.b item for mycobacterium culture.

7.2 Only mycobacterium isolation culture positive pulmonary tuberculosis diagnosis Meet any of 3 item, at least 2 item sputum specimens smear negative and mycobacterium culture meet 4.1.b item.

7.3 Positive diagnosis of pulmonary tuberculosis by molecular biology examination

Meets any of 3 item and 4.2 item

7.4 Positive pulmonary tuberculosis diagnosis by pulmonary histopathological examination

In line with 4.3 item

7.5 Diagnosis of tracheal and bronchial tuberculosis

If one of the following items is met:

A) Have 5 item and meet the requirements of 4.3 item in the pathologic examination of trachea and bronchus;

B) Those with 5 item and etiological examination of trachea and bronchial secretions meeting the requirements of 4.1.a item or

4.1.b item or 4.2 item.

7.6 Diagnosis of tuberculous pleurisy

If one of the following items is met:

A) Having 3 item and meeting the requirements of 4.3 item in pleural hydrothorax or pleural pathology examination;

B) Those that have 3.3 item and pleural effusion and meet the requirements of 4.1.a item or 4.1.b item or 4.2 item.

**7. Source of diagnostic criteria**, detailed testing methods, instruments, procedures, etc:

“WS 288-2017” replacing “WS 288-2008” (Health Industry Standard of China)

<http://wsbz.nhc.gov.cn/wsbzw/upload/StandardLibrary/a97ede2f50ae4235bdd00b6e8e27ae86.pdf>

**Scarlet Fever**

**1. Clinical symptoms**：Related clinical symptoms (omitted).

**2. Contact history:** This disease occurred and was endemic locally. Contact history with scarlet fever patients or with tonsillitis, pharyngitis, otitis media, erysipelas and other streptococcal infection history.

**3. Laboratory Tests:**

3.1 The total number of white blood cells and neutrophils increased.

3.2 The rapid test of group A streptococcus was positive.

3.3 Microscopic examination of the bacteria culture revealed β hemolytic streptococcus.

3.4 The sensitivity test of bacitracin was positive.

3.5 Biochemical identification of streptococcus pyogenes.

3.6 The pharyngeal swab or other lesion secretions were serologically grouped and identified as group A beta hemolytic streptococcus.

**4. Principles of diagnosis**

Comprehensive analysis of clinical symptoms, contact history and laboratory tests, and diagnosis must be based on examination for etiology.

**5. Source of diagnostic criteria**, detailed testing methods, instruments, and procedures, etc:

“WS 282-2008” (Health Industry Standard of China)

<http://wsbz.nhc.gov.cn/wsbzw/upload/StandardLibrary/d32c1474057f42a0ba227aab5e8f8061.pdf>

**Rubella**

**1. Clinical symptoms**：Related clinical symptoms (omitted).

**2. Contact history**: Had no prior exposure to rubella, but had a clear history of contact with a confirmed rubella patient within 14-21 days prior to onset.

**3. Laboratory Tests:**

3.1 Rubella virus isolated from a swab or urine sample, or rubella virus nucleic acid detected.

3.2 Positive serum rubella IgM antibody (not vaccinated rubella attenuated live vaccine within 1 month).

3.3 The titer of serum rubella IgG antibody or rubella hemagglutination-inhibiting antibody in recovery period was more than 4

times higher than that in acute period.

3.4 Acute phase antibody is negative but convalescent phase antibody change to positive.

**4. Principles of diagnosis**

According to clinical manifestations combined with epidemiology, clinical diagnosis could be made, and according to the detection of serum rubella antibody or rubella pathogen detection, cases could be confirmed.

**5. Source of diagnostic criteria**, detailed testing methods, instruments, procedures, etc:

“WS 297-2008” (Health Industry Standard of China)

<http://wsbz.nhc.gov.cn/wsbzw/upload/StandardLibrary/f63275dc3179424790f603062b27991f.pdf>

**Measles**

**1. Clinical symptoms**：Related clinical symptoms (omitted).

**2. Contact history:** History of contact with patients diagnosed with measles 7 -21 days before eruption;

Residence or travel history in measles endemic area 7 to 21 days prior to eruption.

**3. Laboratory Tests:**

3.1 No live attenuated vaccine containing measles components was inoculated within 8 days to 56 days before blood collection,

while measles IgM was positive in serum samples within 28 days after eruption.

3.2 Measles virus nucleic acid positive or isolated from measles virus in a swab or urine sample.

3.3 The titer of measles IgG antibody in the concomitant blood sample was more than 4 times higher than that in the acute phase, or the acute phase was negative and the concomitant phase was positive.

**4. Principles of diagnosis**

According to clinical manifestations combined with epidemiology, clinical diagnosis could be made, and according to the laboratory tests, cases could be confirmed.

**5. Source of diagnostic criteria**, detailed testing methods, instruments, procedures, etc:

“WS 296-2017” replacing “WS 296-2008” (Health Industry Standard of China)

<http://wsbz.nhc.gov.cn/wsbzw/upload/StandardLibrary/823d08570cf04ba6be92118acd9b60f0.pdf>

**Pertussis**

**1. Clinical symptoms**：Related clinical symptoms (omitted).

**2. Epidemiological history:**

Pertussis is prevalent in the specific area. There is a history of close contact with pertussis patients, but no history of vaccination.

**3. Laboratory Tests:**

3.1 The white blood cell count and lymphocyte in peripheral blood increased obviously.

3.2 Bordetella pertussis was isolated from sputum, and nasopharyngeal secretions.

3.3 The increase of serum specific antibody in recovery phase was more than 4 times than that of acute phase.

**4. Principles of diagnosis**

Pertussis cases can be diagnosed according to the epidemiological history, clinical manifestations and laboratory examination results.

**5. Source of diagnostic criteria**, detailed testing methods, instruments, procedures, etc:

“WS 274-2007” (Health Industry Standard of China)

<http://wsbz.nhc.gov.cn/wsbzw/upload/StandardLibrary/a881fd0c453d4b00ae3d2cb422190506.pdf>

**Meningococcal meningitis**

**1. Clinical symptoms**：Related clinical symptoms (omitted).

**2. Contact history:**

Local residents have a history of living or traveling in the epidemic area 10 days before the onset of the disease.

**3. Laboratory Tests:**

3.1 Routine blood

The total white blood cell count and neutrophil count were significantly increased.

3.2 Routine cerebrospinal fluid

The typical change was increased pressure, and the appearance was cloudy rice soup or pus. The number of white blood cells increased obviously, and polynuclear white blood cells increased mainly. Sugar and chloride significantly reduced, protein content increased. However, in the early stage of the course of disease, only the pressure is increased and the appearance is clear, followed by typical changes. The cerebrospinal fluid of patients with outbreak shock is usually clear, and the protein, cell number and sugar are not changed.

3.3 Pathogenic detection

3.3.1 Examination of ecchymosis (spot) tissue fluid and cerebrospinal fluid smear, gram negative renal diplococcus can be found

in polynuclear white blood cells or outside cells.

3.3.2 Neisseria meningitidis was positive in cerebrospinal fluid, blood and tissue fluid of stasis (spot).

3.3.3 The specific nucleic acid of neisseria meningitidis tested positive in cerebrospinal fluid, blood and tissue fluid of petechiae (spots).

3.4 Immunology

3.4.1 The specific polysaccharide antigen of neisseria meningitidis was tested positive for cerebrospinal fluid samples in the acute phase.

3.4.2 Detection of neisseria meningitidis specific IgG antibody in convalescent serum showed a 4-fold or more increase in titer than that in acute phase.

**4. Principles of diagnosis**

The diagnosis of suspected cases and/or clinically diagnosed cases was made according to the epidemiological history, clinical manifestations, blood routine and/or cerebrospinal fluid routine test results.

Diagnosis requires the results of pathogenic or immunological tests of neisseria meningitidis, and further diagnosis of pathogenic clusters in cases with positive pathogenic tests.

**5. Source of diagnostic criteria**, detailed testing methods, instruments, and procedures, etc:

“WS 295-2019” replacing “WS 295-2008” (Health Industry Standard of China)

<http://wsbz.nhc.gov.cn/wsbzw/upload/StandardLibrary/bb3dac1523f24bbf88629b28564001e6.pdf>

**Leprosy**

**1. Clinical symptoms**：Related clinical symptoms (omitted).

**2. Contact history:**

Patients lived in endemic areas and had close contact with untreated leprosy patients.

The relatives, neighbors or colleagues have leprosy patients and have close contact with them before diagnosis and treatment.

**3. Laboratory Tests:**

Routine skin examination showed positive AFB in one or more parts (see URL below).

Histological examination of skin lesions (see URL below)

**4. Principles of diagnosis**

According to the epidemiological history, clinical manifestations, combined with laboratory examination (skin smear examination AFB and tissue biopsy of leprosy specific pathological changes), comprehensive analysis, timely and accurate diagnosis.

**5. Source of diagnostic criteria**, detailed testing methods, instruments, and procedures, etc:

“WS 291-2018” replacing “WS 291-2008” (Health Industry Standard of China)

http://wsbz.nhc.gov.cn/wsbzw/upload/StandardLibrary/f4e46f352cd64e5a8cdc9e22fff96eab.pdf
