# Supplementary figures and images for: Wildfire-related PM2.5 and respiratory transmitted disease among Chinese children and adolescents from 2008 to 2019: A retrospective study
Source: PLoS Med. 2025 Dec 5;22(12):e1004613. doi: 10.1371/journal.pmed.1004613 (PMC12680207; doi:10.1371/journal.pmed.1004613)

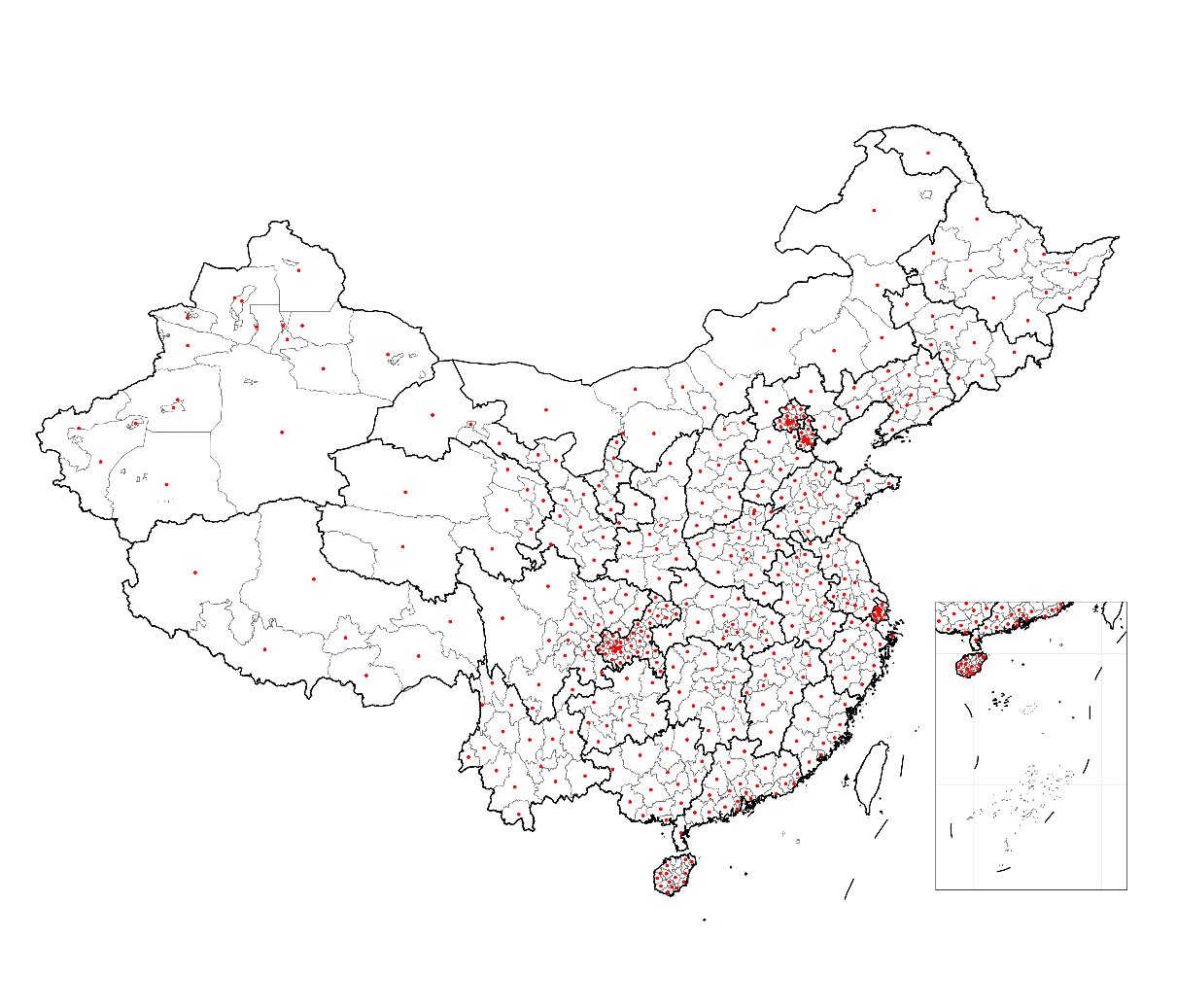

Supplement: S1 Fig — Notes: Spatial boundaries were retrieved from Natural Earth (https://www.naturalearthdata.com/) using the “rnaturalearth” package (https://github.com/ropenscilabs/rnaturalearth). (TIF) [file pmed.1004613.s005.tif]

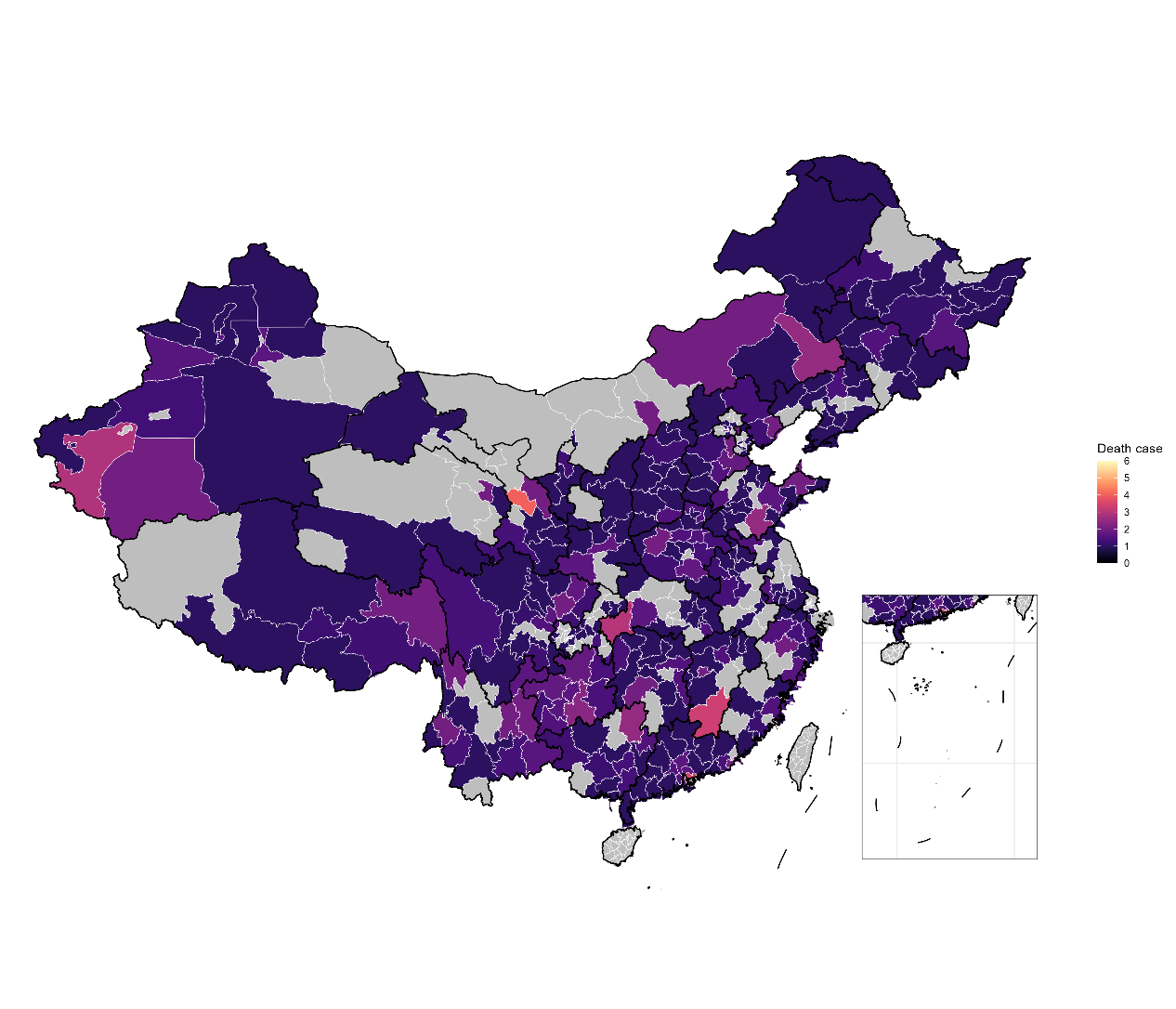

Supplement: S2 Fig — Notes:Spatial boundaries were retrieved from Natural Earth (https://www.naturalearthdata.com/) using the “rnaturalearth” package (https://github.com/ropenscilabs/rnaturalearth). (TIF) [file pmed.1004613.s006.tif]

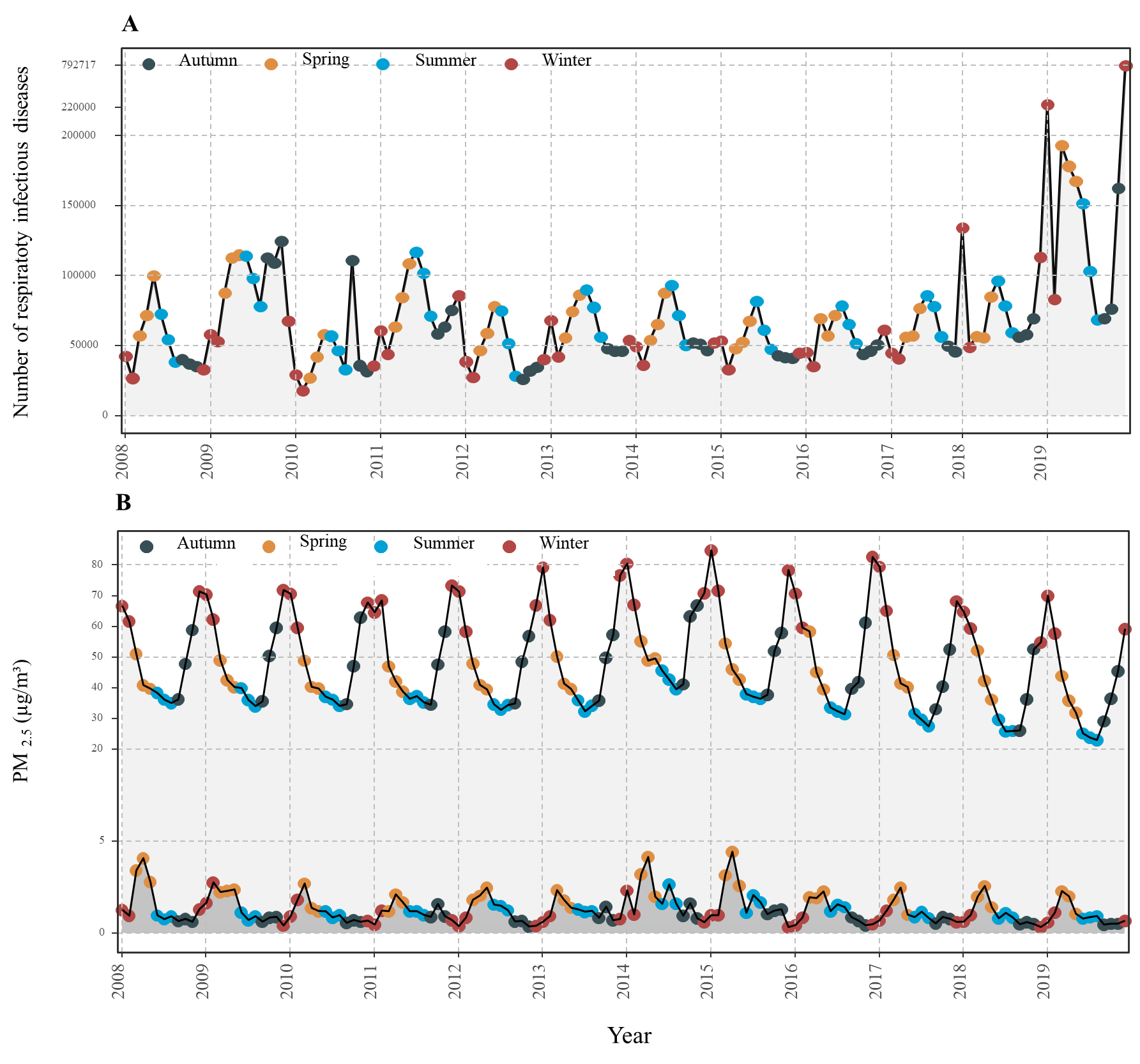

Supplement: S3 Fig — (TIF) [file pmed.1004613.s007.tif]

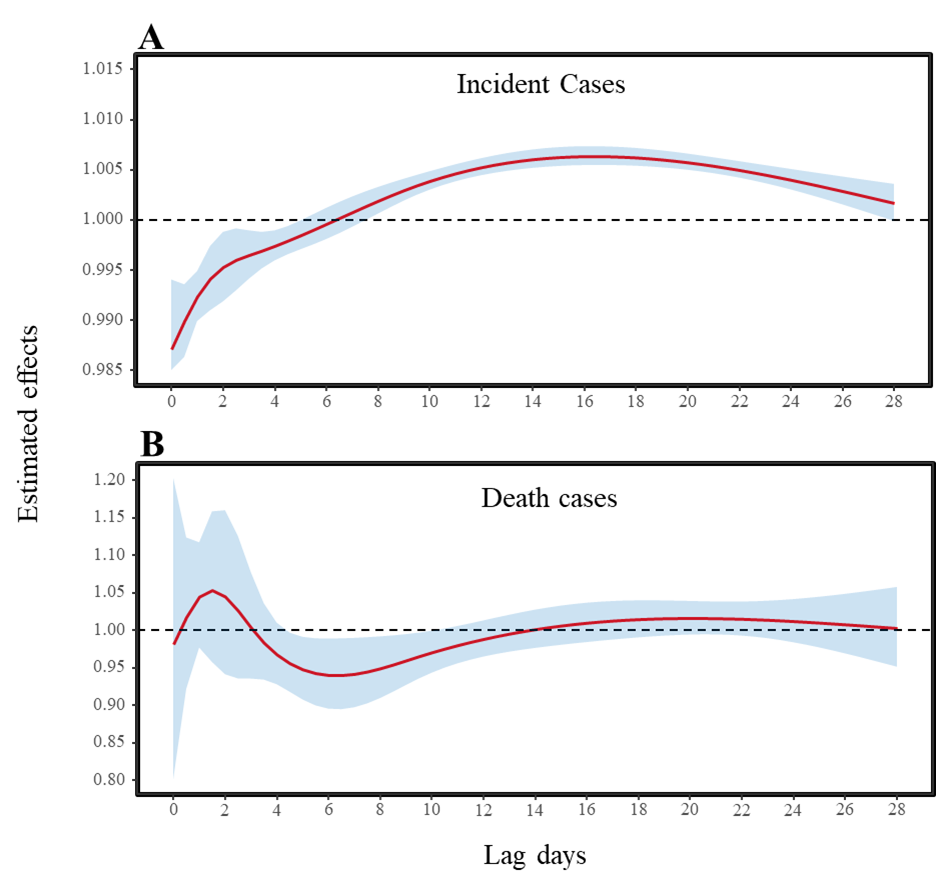

Supplement: S4 Fig — (TIF) [file pmed.1004613.s008.tif]

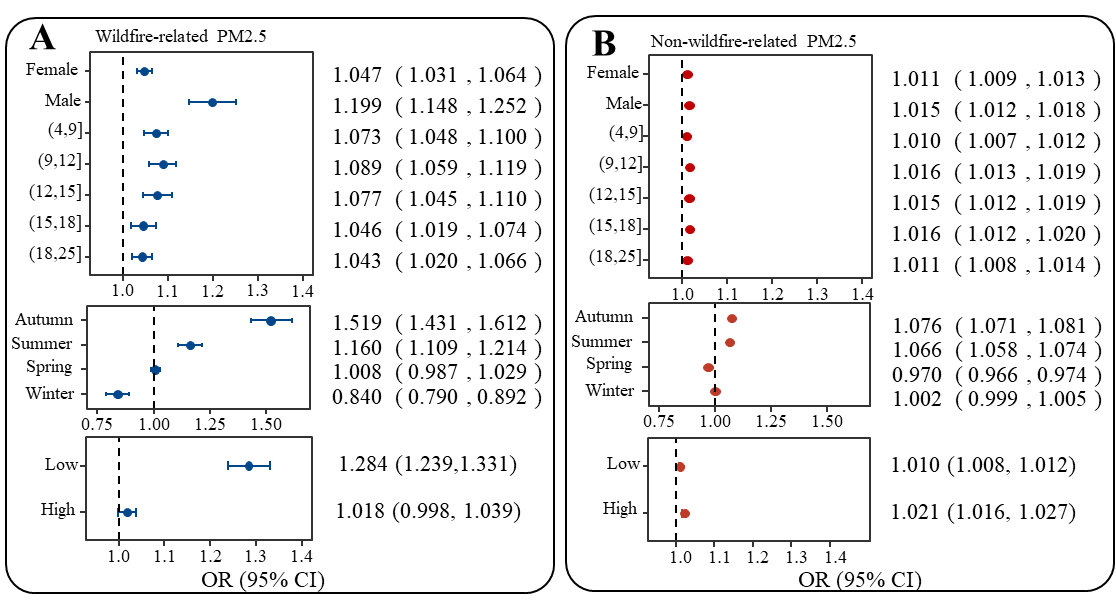

Supplement: S5 Fig — Note: Low refers to areas with yearly wildfire-related PM2.5 concentrations <1.5 μg/m3; High denotes areas with yearly wildfire-related PM2.5 concentrations ≥1.5 μg/m3. Subfigure A presents the estimated association between respiratory transmitted disease and wildfire-related PM2.5 by subgroups, while Subfigure B presents the estimated association between respiratory transmitted diseases and non-wildfire-related PM2.5 by subgroups. (TIF) [file pmed.1004613.s009.tif]

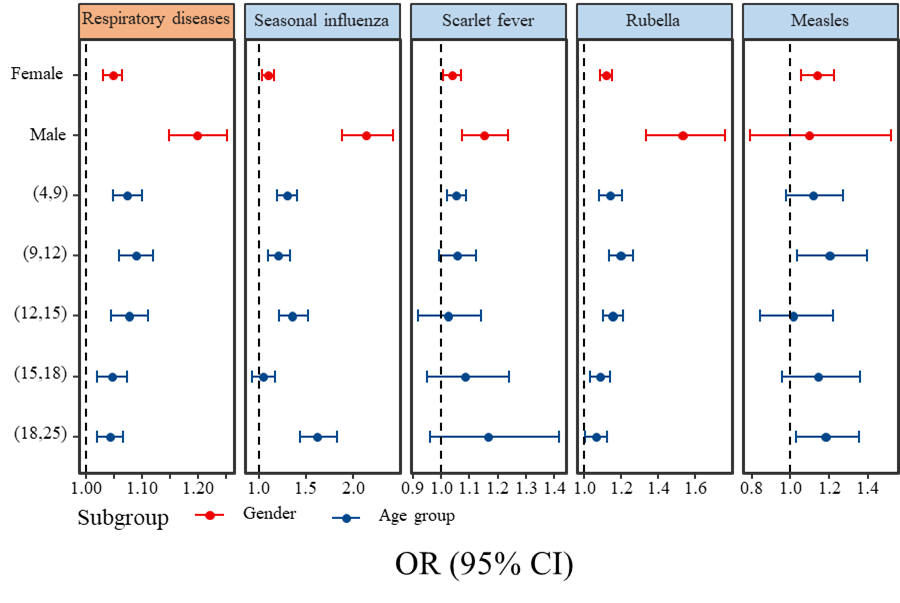

Supplement: S6 Fig — (TIF) [file pmed.1004613.s010.tif]

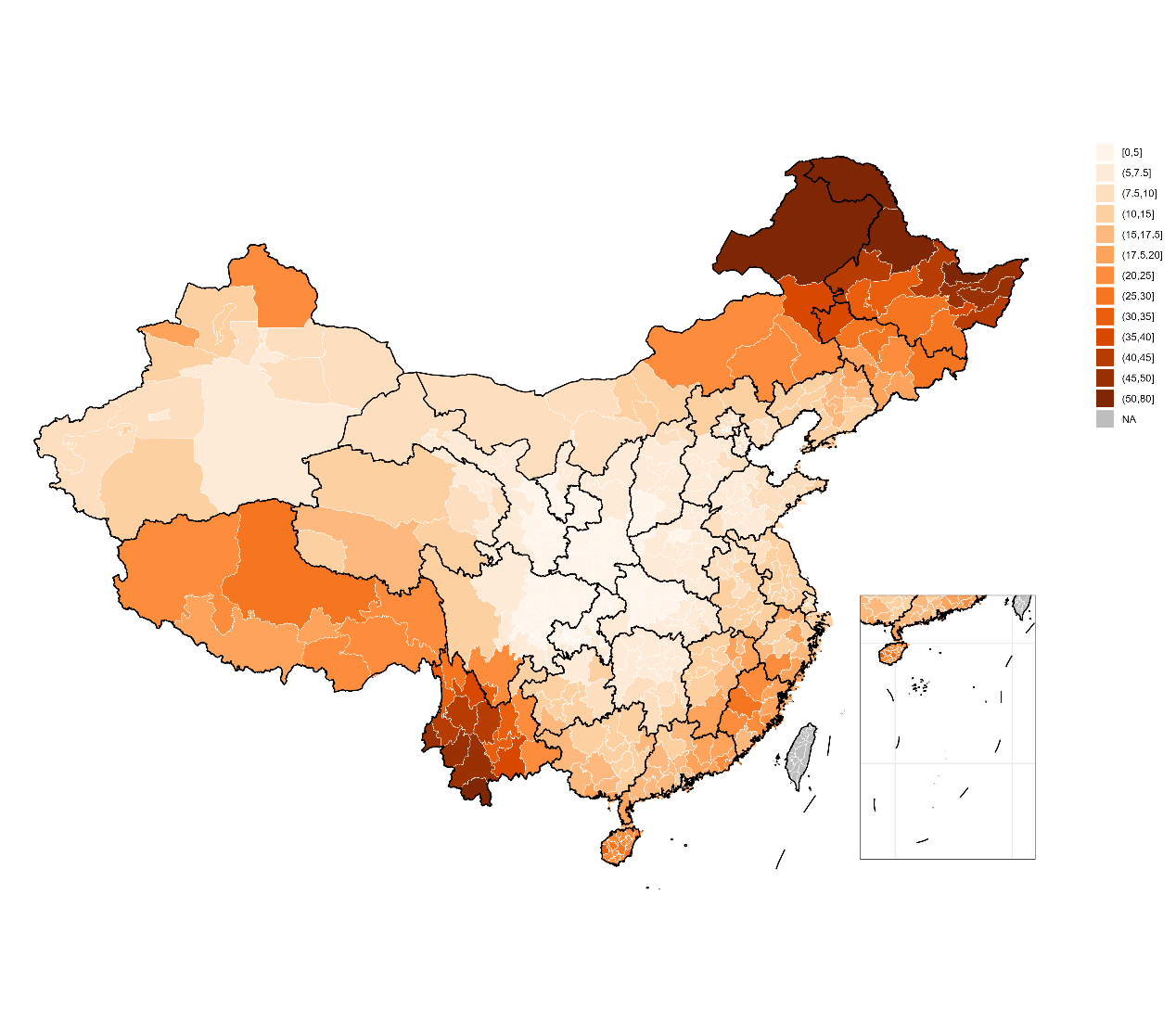

Supplement: S7 Fig — Notes; Spatial boundaries were retrieved from Natural Earth (https://www.naturalearthdata.com/) using the “rnaturalearth” package (https://github.com/ropenscilabs/rnaturalearth). (TIF) [file pmed.1004613.s011.tif]
